# Understanding Pathways into Care-homes using Data (UnPiCD study): a two-part model to estimate inpatient and care-home costs using national linked health and social care data

**DOI:** 10.1186/s12913-024-10675-z

**Published:** 2024-03-05

**Authors:** G. Ciminata, J. K. Burton, T. J Quinn, C. Geue

**Affiliations:** 1 Health Economics and Health Technology Assessment, School of Health & Wellbeing, University of Glasgow, Glasgow, Scotland, U.K.; 2 Academic Geriatric Medicine, School of Cardiovascular & Metabolic Health, University of Glasgow, Glasgow, Scotland, U.K.

**Keywords:** Care-home, Hospital, Routine data, Data linkage, Social care, Older people, Costing-analysis, Two-part model

## Abstract

**Background:**

Pathways into care-homes have been under-researched. Individuals who move-in to a care-home from hospital are clinically distinct from those moving-in from the community. However, it remains unclear whether the source of care-home admission has any implications in term of costs. Our aim was to quantify hospital and care-home costs for individuals newly moving-in to care homes to compare those moving-in from hospital to those moving-in from the community.

**Methods:**

Using routinely-collected national social care and health data we constructed a cohort including people moving into care-homes from hospital and community settings between 01/04/2013-31/03/2015 based on records from the Scottish Care-Home Census (SCHC). Individual-level data were obtained from Scottish Morbidity Records (SMR01/04/50) and death records from National Records of Scotland (NRS). Unit costs were identified from NHS Scotland costs data and care-home costs from the SCHC. We used a two-part model to estimate costs conditional on having incurred positive costs. Additional analyses estimated differences in costs for the one-year period preceding and following care-home admission.

**Results:**

We included 14,877 individuals moving-in to a care-home, 8,472 (57%) from hospital, and 6,405 (43%) from the community. Individuals moving-in to care-homes from the community incurred higher costs at £27,117 (95% CI £ 26,641 to £ 27,594) than those moving-in from hospital with £24,426 (95% CI £ 24,037 to £ 24,814). Hospital costs incurred during the year preceding care-home admission were substantially higher (£8,323 (95% CI£8,168 to £8,477) compared to those incurred after moving-in to care-home (£1,670 (95% CI£1,591 to £1,750).

**Conclusion:**

Individuals moving-in from hospital and community have different needs, and this is reflected in the difference in costs incurred. The reduction in hospital costs in the year after moving-in to a care-home indicates the positive contribution of care-home residency in supporting those with complex needs. These data provide an important contribution to inform capacity planning on care provision for adults with complex needs and the costs of care provision.

**Supplementary Information:**

The online version contains supplementary material available at 10.1186/s12913-024-10675-z.

## Background

In the UK, individuals can move-in to care-homes directly from a hospital admission or from the community. Despite these being everyday lived experiences for individuals and their families, pathways into care-homes have been under-researched. The Understanding Pathways into Care-homes using Data (UnPiCD), study seeks to address this research gap through a programme of data linkage research. Our initial study identified important differences in the characteristics of those moving-in to care-homes directly from hospital, compared to those moving-in from the community, in terms of their level of frailty, morbidity and dependency [1]. In the second phase of work, we have sought to explore whether the source of care-home admission has any implications in term of costs. The theory is that as people moving-in from hospital and community are clinically different that this may be reflected in the healthcare resource used and associated costs incurred. Further understanding of care-home pathways, including knowing whether one group require more resources than the other, may help shaping care service planning more effectively.

Many studies consider disease-specific costs, evaluate changes in policy or assess the cost and cost-effectiveness of interventions within a care-home setting. For example, Meads et al., assesseed a method to measure the experience of people with dementia (Dementia Care Mapping) in care-home settings [2], Romeo and colleagues described a modelling approach for estimating the cost of care-homes in England for a relatively small population consisting of 277 individuals with dementia [3]. Another study carried out by Martin et al. examined links between clinical and other characteristics of people with Alzheimer’s disease living in the community, the likelihood of care home or hospital admission, and associated costs [4]. With regards to policy change, Allan et al. explored differences between private and public prices in the English care-homes market [5], and assessed the impact, over time, of local authorities’ expenditure on the supply of care-homes [6]. A study undertaken by Sahota assessed the direct cost of acute hip fracture in care home residents [7]. Alili et al. conducted an economic evaluation of a care family program for nursing home residents with advanced dementia [8], and Logan et al. assessed a multidomain decision support tool to prevent falls in older people using a trial design approach [9]. These studies were mainly undertaken as economic evaluations.

Studies that specifically estimate the cost of care-home stay (databases and search strategy presented in the supplementary material) are scarce. In addition, given the heterogeneous care-home landscape, such studies ought to be undertaken at national level, so that findings can inform decision making at local/national level. To our knowledge, only one study has attempted to estimate the cost of care-home stays in Scotland; the authors have estimated the contribution of care-home costs towards the overall cost of Atrial Fibrillation (AF) including inpatient, outpatient and prescribing costs. The existing study has estimated global costs using individual-level linked data from Scotland for people with a diagnosis of AF or atrial flutter between 1997 and 2015 [10]. In our current study, we adopt a more inclusive approach without focussing on a specific health condition and differentiating individuals that have moved-in from hospital or community.

Our aim was to quantify hospital and care-home costs for individuals newly moving-in to care homes to compare those moving-in from hospital to those moving-in from the community. Our objective is therefore, within a novel framework, to quantify inpatient and care-home costs, using the Scottish Care-Home Census (SCHC) as a unique data source linked to health data sets. The SCHC is a national dataset, composed of data submitted by care-home staff, collected by the Care Inspectorate (a regulatory body assessing the quality-of-care services in Scotland) and the Scottish government, and analysed by Public Health Scotland (PHS, national statistics provider). The SCHC includes both care-home level data (including weekly charges) and individual resident level data, which can be linked to other national data sources [11]. This overcomes the limitations of NHS data, not fit for purpose for reliably identifying all individuals that live in care-homes [12] and provides a more inclusive representation of care-home residents typically under-represented in epidemiological studies [13]. Studying care-home pathways requires individual-level national data. However, care-home research studies are often confined to single care providers or regions [14, 15]. The care-home level data within SCHC allows, at a national level, to distinguish between different pathways into care-home and associated variation in resource utilisation and costs. The remainder of the paper is organised as follows: description of the context for the costing-analysis, description of cohort and cost data, description of the econometric model and the covariates, results describing cohort characteristics and cost estimates, and discussion including strength and limitations of the study.

## Methods

In the present study we have adopted a comprehensive approach to estimate costs associated with care-home and hospital stay [16]. We included costs associated with hospital admissions, psychiatric admissions and care-home charges, incurred by individuals moving-in into care-home, that are not related to any specific disease.

### Context

In Scotland an adult care home is defined as a 24-hour residential care facility and includes services with and without on-site registered nursing staff. All adult care homes are registered by the national regulator, the Care Inspectorate [17]. Most care homes in Scotland (73.7%) are for older adults (aged 65 years and over). Other services support those with learning disabilities (16.6%), mental health problems (5.1%), physical and sensory impairment (3.2%) and substance misuse problems (1.5%) [18]. Moving-in to a care home can be a temporary arrangement, such as for respite or intermediate care, or for long-term care. Average length of stay in Scotland’s care homes is 1.8 to 2.7 years, with differences between age and sex groups [19]. There is an agreed National Care Home Contract in Scotland annually, by which Local Authorities determine the weekly charge they will pay towards care home fees. However, weekly charges vary significantly across the country [20]. Assessment of an individual’s assets will determine the level of contribution made to the costs of care home placement [21]. In addition, there is a nationally agreed rate for residents assessed as requiring personal care (£233.10 per week in 2023) and nursing care (additional £104.90 per week in 2023), which contributes towards care costs [22].

Care needs are assessed by a Social Worker who will determine the level of care an individual requires, support the financial assessment process and establish eligibility to receive care allowances. This assessment will be shared with care home providers, who will determine if they can meet the needs of the individual concerned. This process may occur while an individual is in hospital or in the community.

### Data

Data were obtained from PHS as part of a wider project that uses routinely collected data to understand pathways into care-home [1]. Annual data on individuals moving-in to care homes for long-term care in Scotland were obtained from the SCHC for the financial years 2013/14, 2014/15 and 2015/16. Because submission to the SCHC is not mandatory, for our study period, we were able to include about ~ 80% of care-home residents. Within the SCHC, distinctions are made between publicly funded (mainly or fully funded by the Local Authority) or privately funded (mainly or fully self-funded) care-home residency and whether individuals receive nursing care or not [23]. The latter distinction indicates whether a resident receives nursing care funding in addition to free personal care funding.

We identified individuals with a first care-home admission in SCHC for the financial year 2013/14, 2014/15 and followed them up until 2016. Individual-level data linkage was then carried out with general acute inpatient and day case records (Scottish Morbidity Records 01; SMR01) [24], mental health inpatient and day case records (Scottish Morbidity Records 04; SMR04) [25], geriatric long-stay records (Scottish Morbidity Records 50; SMR50) [26] and mortality records (National Records for Scotland, NRS) [27].

From the 18,605 individuals identified in SCHC, with a first care-home admission in the financial year 2013/14 (*N* = 9,424) or 2014/15 (*N* = 9,181), we have excluded those there were lost to follow up (Figure S1).

Following the method of classification described in the UnPiCD study (characterising individuals who move-in to a care home from hospital and compare with those moving-in from the community) [1], in our final cohort (*N* = 16,931), we classified individuals according to their source of care-home admission: “from hospital” (*N* = 8,472), “from community” (*N* = 6,405), and “from another care-home” (*N* = 2,054). In the present study we focused on the first two groups of people.

### Costing

Costs for care-home stay were obtained from SCHC, for the years 2013/14 to 2015/16. The Census includes unit costs on weekly charges for long stay residents in care-homes, classified according to whether the residency is publicly funded or privately funded and whether individuals receive nursing care or not. Informed by the National SCHC methodology document, we reviewed costs that were <£300 or >£1500 [23]. Where data were missing or implausibly low, we calculated weekly mean care-home charges for each category of funding and nursing status to apply to missing/low-cost records. The average was calculated excluding charges >£1,500, as these were from highly specialist services and not representative of most care-home charges. From 905 care-homes having individuals moving-in during the census year 2013/14, we had to calculate average weekly charges for 168 cases of funding and nursing status. For the census year 2014/15, accounting for 847 care-homes, the average weekly charges were calculated for 139 cases.

Inpatient and mental health unit costs were obtained from Specialty Group Costs, PHS, for the years 2013 to 2016 [28]. Specialty group costs for inpatients in all specialties were linked to SMR01 and SMR50. Similarly, specialty group costs for mental health (including unit costs for geriatric long stay, young chronic sick, psychiatry and learning disabilities) were linked to SMR04. Costs data sources and descriptions are summarised in Table 1. We combined care-home and hospital costs to estimate the overall hospital admission and care-home costs per person per year incurred by people moving-in to a care-home. We also estimated care-home and hospital costs as separate cost components. In addition, to test the hypothesis that hospital costs reduce after moving-in to a care-home, we estimated average hospital costs during the year preceding and the year following care-home admission for individuals that were admitted to hospital.

**Table 1:** Cost data sources and descriptions

Cost	Source	Description
Care home stay	Scottish Care-Home Census	Unit costs, for the years 2013/14 to 2015/16, on weekly charges for long stay residents in care-homes, classified according to whether the residency is publicly funded or privately funded and whether individuals receive nursing care or not
Inpatient	Public Health Scotland, Specialty Group Costs - Inpatients	Specialty Group Costs, for the years 2013 to 2016, in all specialties linked to general acute inpatient and day case records (SMR01) and geriatric long-stay records (SMR50)
Mental health	Public Health Scotland, Specialty Group Costs - Inpatients	Specialty Group Costs (including unit costs for geriatric long stay, young chronic sick, psychiatry and learning disabilities), for the years 2013 to 2016, linked to mental health inpatient and day case records (SMR04)

### Econometric model

In order to account for the skewed nature of cost data and a significant proportion of zero-cost observations (for individuals who have not used any healthcare resources in a given time period), a two-part model was used [29]. In the first part of the model, the probability of incurring costs in a given time period was estimated using a probit model.

This was followed by a generalised linear model (GLM) with a log link and gamma distribution (Equation S1 and S2) to estimate costs conditional on having incurred positive costs. in the second modelling part, specifying a log link and gamma distribution (Equation S1 and S2). Model selection was based on the Akaike information criterion (AIC). GLM reported the lowest AIC, indicating the best fit for our data.

### Covariates

The two-part model was adjusted for what are considered to be main confounders that have an effect on costs incurred by individuals during their care-home and hospital stay: route of care-home admission, age, sex, year of admission, main client group, frailty risk score and mortality. In addition to controlling for care-home route of admission, we adjusted our model for age and sex, as we have assumed, particularly in a care-home setting, cost variations in age and between men and women.

We also assumed variation in healthcare utilisation and associated costs between client groups, classified as older adults, individuals with learning disabilities and other adults (including mental health problems, physical disabilities and sensory impairment). Further, as we anticipate cost differences between individuals with different levels of frailty, we adjusted our model for the hospital frailty risk score, where < 5 indicates low risk, 5–15 intermediate risk, and > 15 high risk [30]. Two interaction terms were included in the econometric model to account for a relationship of direct proportionality between age and mortality, and age and frailty.

## Results

### Cohort characteristics

Of the 14,877 individuals admitted to a care-home, 8,472 (57%) move-in from hospital, and 6,405 (43%) from the community. Most care-home residents were females (~ 66%), in the age group of 80–99 years (~ 72%), mostly represented by the “older adults” client group (~ 96%) (Table 2). We have also found that most individuals with learning disabilities (~ 80%) and those classified as “other adult” (~ 70%) were amongst the youngest care-home residents (age group < 60).

**Table 2:** Baseline characteristics of people moving-in from hospital and community

	Source of care home admission		
	All (14,877)	Hospital (8,472)	Community (6,405)
Sex			
Male	5,023 (33.8)	3,015 (35.6)	2,008 (31.4)
Female	9,854 (66.2)	5,457 (64.4)	4,397 (68.6)
Age			
< 60	635 (4.3)	262 (3.1)	373 (5.8)
60–69	664 (4.5)	424 (5.0)	240 (3.8)
70–79	2,680 (18.0)	1,535 (18.1)	1,145 (17.9)
80–89	6,980 (46.9)	4,016 (47.4)	2,964 (46.3)
90–99	3,779 (25.4)	2,156 (25.5)	1,623 (25.3)
> 100	139 (0.9)	79 (0.9)	60 (0.9)
Main client group			
Older adult	14,333 (96.3)	8,257 (97.4)	6,076 (94.9)
Learning disabilities	183 (1.2)	56 (0.7)	127 (2.0)
Other adult	361 (2.4)	159 (1.9)	202 (3.1)
Frailty			
Low risk (< 5)	4,898 (32.9)	1,383 (16.3)	3,515 (54.9)
Intermediate risk (5–15)	6,109 (41.1)	4,103 (48.4)	2,006 (31.3)
High risk (> 15)	3,870 (26.0)	2,986 (35.3)	884 (13.8)
Died during follow up			
All	6,265	3,988	2,277
2013/2014	1,146 (18.3)	818 (20.5)	328 (14.4)
2014/2015	2,660 (42.5)	1,742 (43.7)	918 (40.3)
2015/2016	2,459 (39.2)	1,428 (35.8)	1,031 (45.3)
Time to death or end of follow up			
Range	0–1,095	0–1,095	0–1,095
Mean (SD)	570 (290.28)	549 (294.74)	596 (282.31)
Median (IQR)	568 (380–800)	548 (356–783)	594 (401–828)
Funding			
All	14,767	8,412	6,355
Public with nursing	6,575 (44.5)	4,241 (50.4)	2,334 (36.7)
Public without nursing	3,585 (24.3)	1,583 (18.8)	2,002 (31.5)
Private with nursing	3,031 (20.5)	1,901 (22.6)	1,130 (17.8)
Private without nursing	1,576 (10.7)	687 (8.2)	889 (14.0)
Switch funding	213 (1.4)	108 (1.3)	105 (1.7)
From public to private (with nursing)	66 (31.0)	40 (37.0)	26 (24.8)
From public to private (without nursing)	65 (30.5)	25 (23.2)	40 (38.1)
From private to public (with nursing)	47 (22.1)	24 (22.2)	23 (21.9)
From private to public (without nursing)	35 (16.4)	19 (17.6)	16 (15.2)

Individuals moving-in from hospital appear to be sicker than individuals moving-in from hospital. This is reflected in the proportion of individuals with a high risk of frailty (35.3%) compared to those moving -in from community (13.8%), and the proportion of individuals that have died during follow up (47.1% from hospital, 35.6% from community) Regardless of the route of care-home admission, less the 2% of individuals switched between source of fundings.

### Modelling results and cost estimates

#### Care-home and hospital cost

**Fig. 1:**
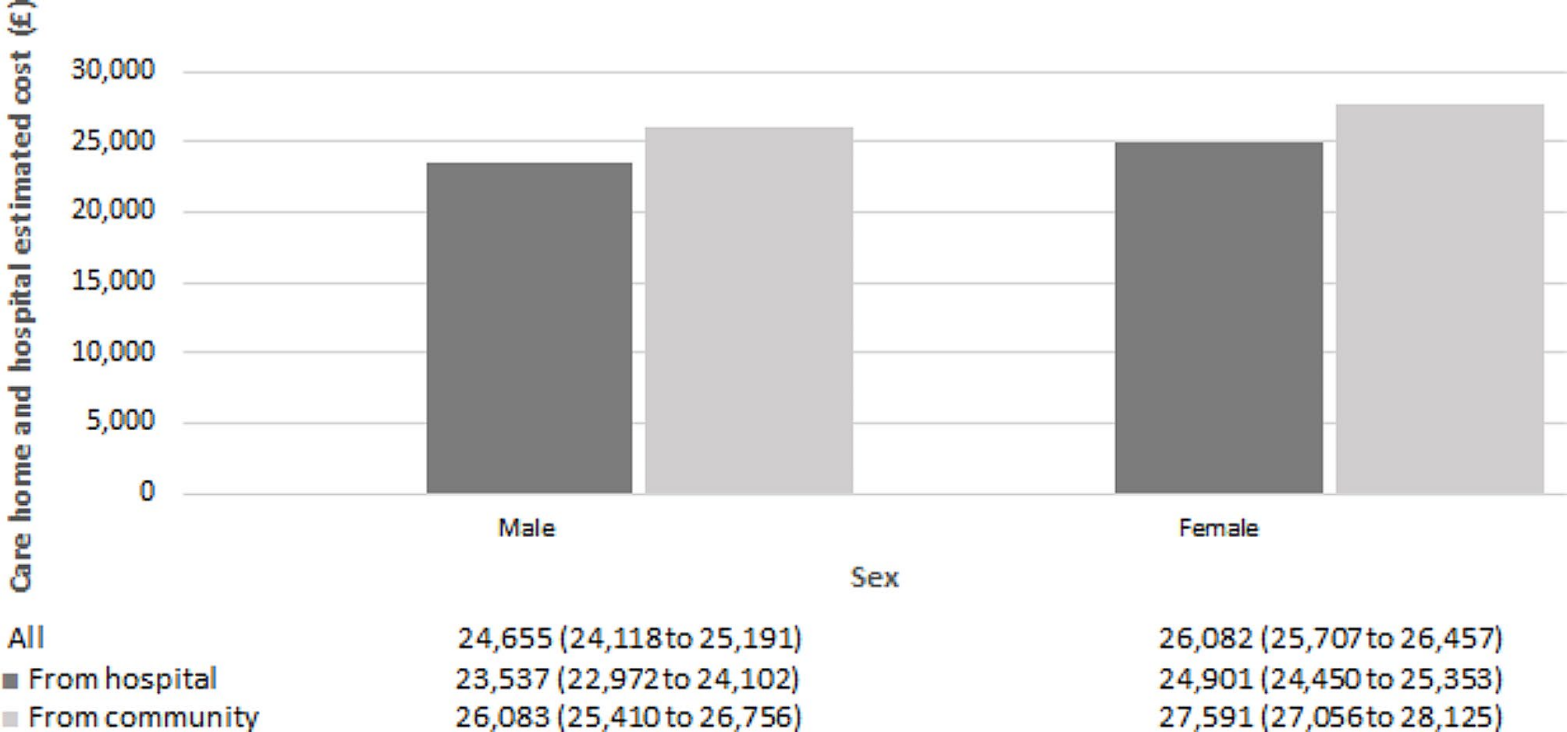
Average annual care-home and hospital cost per person by sex

**Fig. 2:**
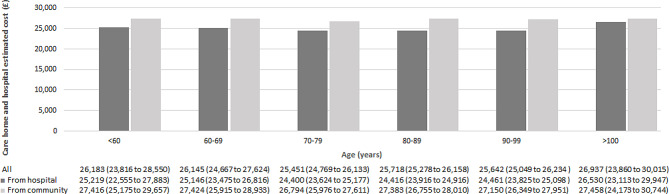
Average annual care-home and hospital cost per person by age

**Fig. 3:**
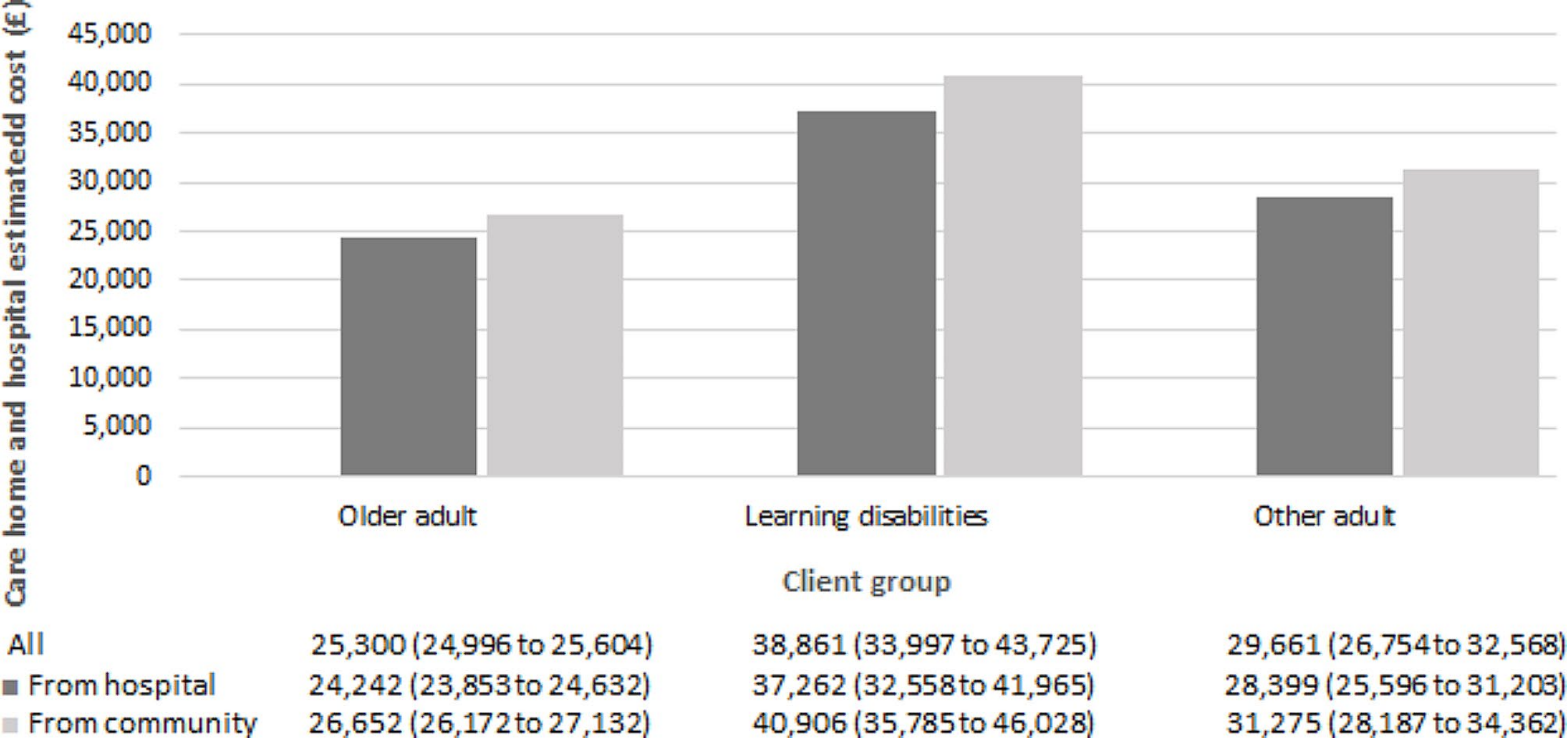
Average annual care-home and hospital cost per person by client group

**Fig. 4:**
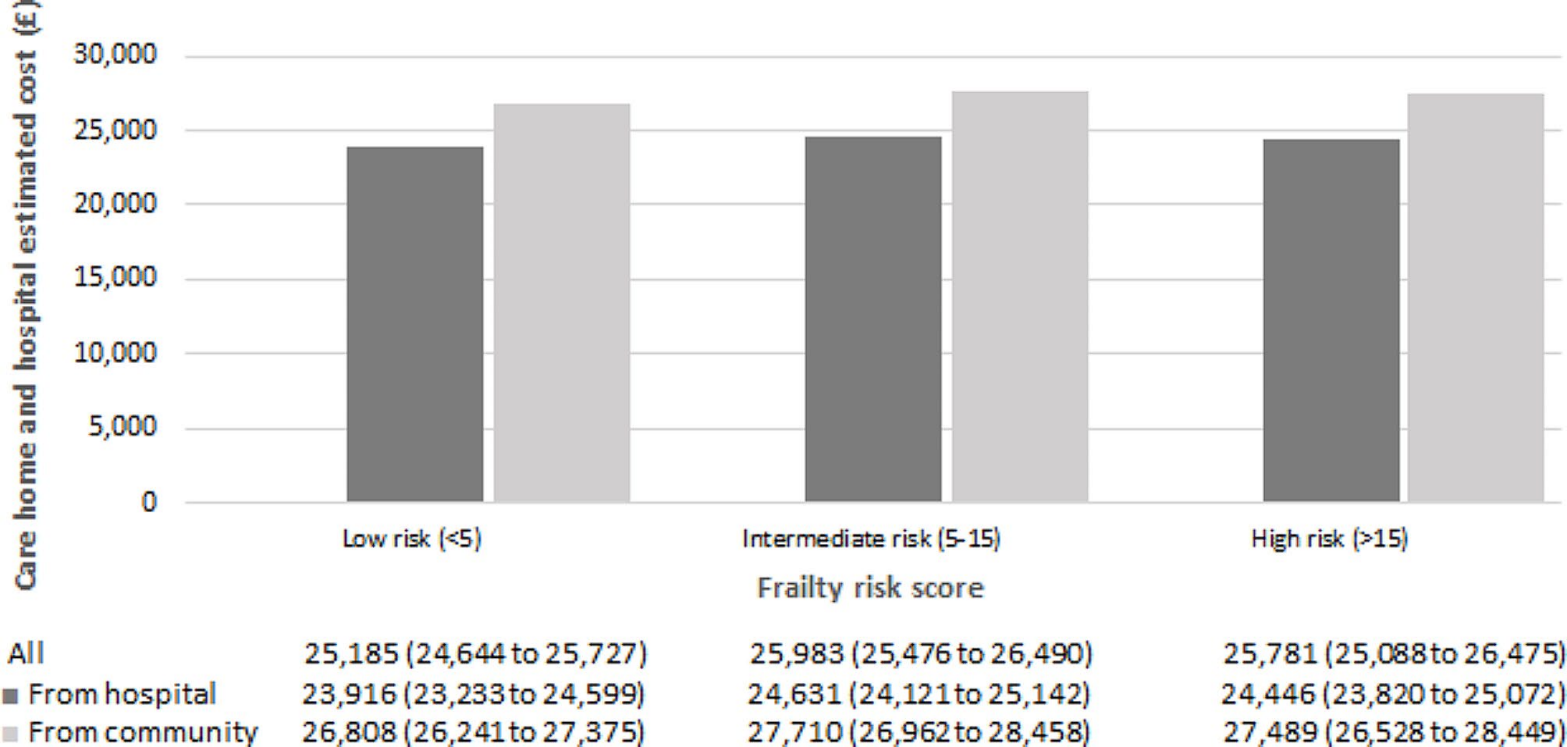
Average annual care-home and hospital cost per person by frailty risk score

Regression results for both modelling parts are presented in the supplementary material (Table S1). No difference in the probability of utilising healthcare services was found between groups of care-home residents. We do however observe a statistically significant increase in care-home and hospital costs in the second modelling part, estimating costs conditional on having incurred positive costs. Individuals moving-in from community and from hospital incurred £27,117 (95% CI £ 26,641 to £ 27,594) and £24,426 (95% CI £ 24,037 to £ 24,814) respectively, per person per year. The cost estimates obtained from the unadjusted model were similar to those obtained from the adjusted model. Estimated total costs by sex, age, client group and frailty risk score appear to be higher for individuals moving-in from community (Figs. 1, 2, 3 and 4). Cost differences appear to be negligible across sex, age, and frailty score. Individuals with learning disabilities appear to incur the highest care-home and hospital costs per person with £38,861 (95% CI £ 33,997 to £ 43,725). These estimates differed for those moving-in from community (£40,906 (95% CI £ 35,785 to £ 46,028) compared to those moving-in from hospital (£37,262 (95% CI £ 32,558 to £ 41,965).

### Care-home cost

Statistically significant increase in care-home costs were observed when care-home costs were estimated as a separate cost component (Table S2) The cost estimates (Figures S2 to S5), were in line with those estimated when combining care-home and hospital costs.

### Hospital cost

When hospital costs were estimates as a separate cost component, no differences between groups were observed in the use of healthcare services and associated costs (Table S3). The estimated hospital costs appeared to be slightly higher for individuals moving-in from hospital (£2,114 (95% CI £ 1,995 to £ 2,233) than for those moving-in from community (£1,787 (95% CI £ 1,664 to £ 1,910). The estimated hospital costs by age, sex, client group and frailty risk score appear to be higher for individuals moving-in from hospital (Figures S6 to S9). While hospital cost differences appear to be negligible between males and females, a negative gradient between age and costs indicated decreasing costs as the cohort ages. Similarly, a positive gradient indicated increasing costs associated with the increased risk of frailty. Individuals with mental health problems, physical disabilities and sensory impairment appear to incur the highest hospital costs per person with £2,888 (95% CI £ 1,675 to £ 3,628). These estimates were higher for those moving-in from hospital (£3,094 (95% CI £ 1,820 to £ 3,975) compared to those moving-in from community (£2,647 (95% CI £ 1,619 to £ 3,674).

### Hospital cost for the year preceding and the year after care-home admission

Additional analyses showed that the use of healthcare services and associated costs in the year preceding care-home admission decreased significantly for individuals moving-in from community, compared to those moving in from hospital (Table S4); but no differences were observed in year after care-home admission (Table S5). In the year preceding care-home admission, individuals moving-in from hospital, incurred considerably higher costs (£11,661 (95% CI£11,436 to £11,885) compared to those moving-in from community (£3,359 (95% CI£3,198 to £3,520) (Figure S10).

## Discussion

This study has provided a novel framework for quantifying inpatient and care-home costs for individuals moving-in to a care-home from a hospital and those moving-in from a community setting, and highlighted the importance of defining pathways into care-homes using real-world observational data. While interventions in care-home settings have previously been evaluated using a trial design [9], such a study design is not feasible when defining pathways into care-homes and estimating associated costs.

We found that for the combined cost including care-home and hospital stay, with care-home costs being the main cost driver, individuals moving-in from the community tend to incur higher follow-up costs. Presumably, individuals moving-in from hospital are sicker and therefore more likely to be readmitted to hospital, hence incurring higher inpatient cost but lower cost for care-home stay. This is evident when hospitalisation is the only cost component factored into the cost estimation, where individuals moving-in from hospital appear to incur higher cost compared to those moving-in from community. This is further substantiated by the proportion of individuals who have died during follow up, as those who were moving-in from hospital, dying sooner, would have a shorter care-home length of stay and would therefore incur lower care-home costs.

Cost for care-home and hospitalisation while in care-home were expected to increase with age. The assumption is that older age groups make greater use of healthcare services than younger age groups and therefore incur higher costs. However, in our adjusted model, the effect that age has on combined hospital and care-home costs, appears to be fairly consistent across age groups. Nevertheless, individuals with learning disabilities, of whom ~ 80% fall within the younger age group, have been found to incur the highest overall healthcare cost. This not only indicates that healthcare expenditure is multifactorial depending on different aspects including age, but that care-home costs do not only concern older people. People living in care-homes have markedly different life expectancy than those living elsewhere in the community, particularly at younger ages [19], reflecting their complex care needs signalled by requiring residential long-term care.

Our findings are in line with existing evidence on individuals with learning disabilities. A studycarried out by Xiao et al., assesse quality outcomes in adult learning disability residential care in the UK, indicated that while residential care home annual fees are now approaching £34,000, for individuals with learning disabilities the annual fees average over £75,000 in many cases [31].

People living in care homes are recognised to use secondary care services, including emergency admissions and Emergency Department attendances [32]. There has been significant interest in the UK [33–35] and internationally [36] on how to support residents within the care-home setting to reduce demand for unscheduled care use. Our findings, in terms of reduced hospital costs in the year after moving-in to a care home, add further weight to the growing evidence around the positive contribution care homes can provide in supporting individuals with complex needs to receive care in a familiar, homely setting [37, 38].

To date, and to our knowledge, only one study has attempted to quantify the cost of care-home stays in Scotland for people with AF [10]. This previous study, combined several routinely collected administrative datasets from Scotland, including care-home utilisation. In our study, however, we have adopted a more comprehensive approach without focussing on specific health conditions and differentiating individuals that have been moving-in from hospital or community.

We have shown, how data linkage can be used to estimate costs associated with care-home and hospital stay. The availability of more contemporary data, prescribing costs, primary and community care cost, in addition to a longer follow-up, could help us shape our future research to address additional important questions: (a) How do costs for individuals moving-in from hospital and those moving-in from the community evolve over time; (b) How do costs relate to primary and community care utilisation and prescribing effect hospital and care home costs for individuals moving-in from hospital and those moving-in from the community.

### Strength and limitations

As Scotland offers a robust record linkage system, where administrative patient-level health data are routinely collected, we were able to link routinely collected social care data with health data and estimate costs that are inclusive of the care-home population in Scotland.

This, coupled with a relatively large sample size, has allowed us to demonstrate the feasibility of cross-sectoral data linkage, where data already submitted by care home staff and repurposed for research can be used in a novel, efficient and timely manner to address questions of public, professional and political interest; thus, providing an important contribution to inform capacity planning on care provision for adults with complex needs and the costs of care provision.

However, we acknowledge a series of limitations. Firstly, in addition to practical challenges related to the structure of the data and the process of data linkage, there were limitations inherent to the nature of administrative data, such as missing records or incomplete data. For instance, in the UnPiCD study (characterising individuals who move-in to a care home from hospital and compare with those moving-in from the community) ~ 7% of the records underwent manual review, resulting in 1.0% of these records being removed as could not be indexed to the Community Health Index (CHI), a number used as the identifier for linkage [1]. Further, because submission to the SCHC is not mandatory, not all care-homes submit their resident-level data, and therefore only about ~ 80% of care-home residents, for our study period, were included in this study [1, 23].

Secondly, we were not able to include unit costs for prescribing held in the Prescribing Information System (PIS), a database that includes prescribing records for all medicines and their associated costs, which are prescribed and dispensed by community pharmacies, dispensing doctors and a small number of specialist appliance suppliers [39]. For our cohort, the data were not available beyond medication counts. Nevertheless, the cost of prescribing tends to have a little impact on overall healthcare costs, and we believe that we have captured the main cost components pertaining to people moving-in to a care-home [10]. In addition, we could not include data on primary and community care cost as these were not available for linkage at a national level.

Thirdly, while we believe that we had sufficient follow-up time for capturing all relevant costs, a longer-follow up may have allowed us to obtain more robust and contemporary cost estimates for all relevant care settings. Finally, the scarcity of studies on the estimation of costs of care-home stay in the UK, did not allowed us to draw robust comparisons with existing evidence.

## Conclusions

We have shown the potential and the challenges of linking social care and health data to estimate costs associated with care-home and hospital stay. We acknowledge that individuals moving-in from hospital and community have different needs. The reduction in hospital costs in the year after moving-in to a care-home indicates the positive contribution of care-home residency in supporting those with complex needs. Further understanding of care-home pathways coupled with recognizing that complex needs drive the cost of care-home stay, may help shaping future health and social care policies aimed at supporting a better plan for healthcare delivery.

### Electronic supplementary material

Below is the link to the electronic supplementary material.

Supplementary Material 1

## Data Availability

All data underlying the analyses are confidential and subject to disclosure control. Data can only be obtained through application to Information Services Division (ISD) via the Public Benefit and Privacy Panel (PBPP), and so are not publicly available.

## Abbreviations

UnPiCD: Understanding Pathways into Care-homes using project Data
SCHC: Scottish Care-Home Census
SMR: Scottish Morbidity Records
NRS: National Records of Scotland
AF: Atrial Fibrillation
PHS: Public Health Scotland
GLM: Generalised Linear Model
AIC: Akaike Information Criterion
