# Supplementary material for: Understanding Pathways into Care-homes using Data (UnPiCD study): a two-part model to estimate inpatient and care-home costs using national linked health and social care data
Source: BMC Health Serv Res. 2024 Mar 5;24:281. doi: 10.1186/s12913-024-10675-z (PMC10916167; doi:10.1186/s12913-024-10675-z)
Supplement: Supplementary file 1 — Supplementary Material 1 [file 12913_2024_10675_MOESM1_ESM.docx]

**SUPPLEMENTARY MATERIAL**

**Literature search strategy**

The search was carried out in Medline (Ovid) to identify studies that estimate the cost of care-home stay. Below are the search terms used for the search strategy:

1. Homes for the Aged/

2. Nursing Home/

3. Residential Home/

4. Care home/

5. Care-home/

6. 1 or 2 or 3 or 4 or 5

7. Economics/

8. cost analysis*mp.

9. 7 or 8

10. United Kingdom/

11. UK/

12. 10 or 11

13. 6 and 9 and 12

**Figure S1. Cohort identification and data extraction**

**Equation S1. Probability of healthcare utilisation**

$$\Pr\left( HCE_{i}>0 \right)=\alpha+\beta_{1}\sum_{c=2}^{2} C_{ci}+ \beta_{2}Y_{i}+ \beta_{3}S_{i}+\beta_{4}\sum_{a=2}^{6} A_{ai}+\beta_{5}\sum_{m=2}^{3} M_{mi}$$

$${+\beta_{6}\sum_{f=2}^{3} F_{fi}+\beta}_{7}D_{di}+\beta_{8}\left( \beta_{4}\sum_{a=2}^{6} A_{ai}*{\beta_{7}D}_{di} \right)+\beta_{9}\left( \beta_{6}\sum_{f=2}^{3} F_{fi}*\beta_{4}\sum_{a=2}^{6} A_{ai} \right) + u_{i}$$

Where: C is route of care home admission (reference: from hospital),Y year of admission; S is sex (reference: male); A is age at the time of admission (reference: 80-89 age group); M is the main client group (reference: older adult); F is the frailty risk score (reference: low risk); D is mortality during two-year follow-up; 𝑢𝑖 is the error term for patient i at time t.

**Equation S2. Cost estimation**

$$E\left[ HC \right]=g(x\beta)$$

Where xβ is the linear predictor for HCE

**CARE HOME AND HOSPITAL COST**

**Table S1. Regression results and interactions for care-home and hospital cost**

|  | Probability |  | Cost ratios |  |
| --- | --- | --- | --- | --- |
|  | (First modelling part) |  | (Second modelling part) |  |
| Covariates | Coefficient (95% CI) | SE | Coefficient (95% CI) | SE |
| **Cohort** |  |  |  |  |
| From hospital | Reference |  |  |  |
| From community | -0.006 (-0.043 to 0.031) | 0.019 | 0.081 (0.058 to 0.104) | 0.012 |
| **Year (cost incurred)** |  |  |  |  |
| 2013/2014 | Reference |  |  |  |
| 2015/2016 | 1.364 (1.293 to 1.434) | 0.036 | -0.420 (-0.441 to -0.398) | 0.011 |
| **Sex** |  |  |  |  |
| Male | Reference |  |  |  |
| Female | -0.007 (-0.045 to 0.030) | 0.019 | 0.059 (0.036 to 0.082) | 0.012 |
| **Age** |  |  |  |  |
| 80-89 | Reference |  |  |  |
| <60 | -0.029 (-0.156 to 0.098) | 0.065 | -0.031 (-0.120 to 0.058) | 0.045 |
| 60-69 | 0.009 (-0.101 to 0.120) | 0.057 | -0.077 (-0.143 to -0.010) | 0.034 |
| 70-79 | 0.011 (-0.058 to 0.080) | 0.035 | -0.045 (-0.090 to 0.000) | 0.023 |
| 90-99 | -0.016 (-0.088 to 0.055) | 0.037 | -0.010 (-0.056 to 0.037) | 0.024 |
| >100 | -0.371 (-0.581 to -0.161) | 0.107 | 0.037 (-0.143 to 0.218) | 0.092 |
| **Main client group** |  |  |  |  |
| Older adult | Reference |  |  |  |
| Learning disabilities | -0.086 (-0.233 to 0.061) | 0.075 | 0.462 (0.349 to 0.575) | 0.058 |
| Other adult | 0.099 (-0.036 to 0.235) | 0.069 | 0.122 (0.035 to 0.210) | 0.045 |
| **Frailty risk score** |  |  |  |  |
| Low risk (<5) | Reference |  |  |  |
| Intermediate risk (5-15) | 0.005 (-0.053 to 0.062) | 0.029 | 0.010 (-0.027 to 0.047) | 0.019 |
| High risk (>15) | -0.017 (-0.082 to 0.049) | 0.034 | 0.001 (-0.039 to 0.042) | 0.021 |
| **Died during follow up** |  |  |  |  |
| Alive | Reference |  |  |  |
| Dead | 1.013 (0.952 to 1.074) | 0.031 | -0.590 (-0.622 to -0.558) | 0.016 |
| **Interaction: age (year) – Mortality** | | | | |
| 80-89 | Reference |  |  |  |
| <60 | -0.115 (-0.494 to 0.265) | 0.194 | 0.071 (-0.121 to 0.262) | 0.098 |
| 60-69 | 0.328 (0.039 to 0.616) | 0.147 | 0.180 (0.057 to 0.303) | 0.063 |
| 70-79 | -0.061 (-0.182 to 0.059) | 0.061 | 0.011 (-0.052 to 0.073) | 0.032 |
| 90-99 | 0.030 (-0.068 to 0.128) | 0.050 | -0.029 (-0.080 to 0.023) | 0.026 |
| >100 | 0.197 (-0.226 to 0.620) | 0.216 | 0.017 (-0.202 to 0.236) | 0.112 |
| **Interaction: age (year) - Frailty score, intermediate risk (5-15)** | |  |  |  |
| 80-89 | Reference |  |  |  |
| <60 | 0.288 (0.093 to 0.482) | 0.099 | -0.028 (-0.157 to 0.101) | 0.066 |
| 60-69 | 0.005 (-0.162 to 0.172) | 0.085 | 0.008 (-0.090 to 0.106) | 0.050 |
| 70-79 | 0.031 (-0.071 to 0.133) | 0.052 | 0.021 (-0.042 to 0.084) | 0.032 |
| 90-99 | 0.031 (-0.068 to 0.130) | 0.051 | 0.013 (-0.048 to 0.074) | 0.031 |
| >100 | 0.534 (0.160 to 0.908) | 0.191 | 0.042 (-0.209 to 0.293) | 0.128 |
| **Interaction: age (year) - Frailty score, high risk (>15)** | | | | |
| 80-89 | Reference |  |  |  |
| <60 | 0.321 (-0.015 to 0.657) | 0.171 | -0.045 (-0.238 to 0.147) | 0.098 |
| 60-69 | 0.045 (-0.166 to 0.256) | 0.108 | -0.022 (-0.150 to 0.106) | 0.065 |
| 70-79 | 0.078 (-0.042 to 0.198) | 0.061 | 0.048 (-0.027 to 0.122) | 0.038 |
| 90-99 | 0.007 (-0.104 to 0.118) | 0.057 | 0.034 (-0.034 to 0.102) | 0.035 |
| >100 | 0.454 (-0.122 to 1.030) | 0.294 | -0.047 (-0.357 to 0.263) | 0.158 |

**CARE-HOME COST**

**Table S2. Regression results and interactions for care-home cost**

|  | Probability |  | Cost ratios |  |
| --- | --- | --- | --- | --- |
|  | (First modelling part) |  | (Second modelling part) |  |
| Covariates | Coefficient (95% CI) | SE | Coefficient (95% CI) | SE |
| **Cohort** |  |  |  |  |
| From hospital | Reference |  |  |  |
| From community | -0.013 (-0.038 to 0.012) | 0.013 | 0.095 (0.073 to 0.118) | 0.012 |
| **Year (cost incurred)** |  |  |  |  |
| 2013/2014 | Reference |  |  |  |
| 2015/2016 | 0.895 (0.823 to 0.967) | 0.037 | -0.265 (-0.285 to -0.245) | 0.010 |
| **Sex** |  |  |  |  |
| Male | Reference |  |  |  |
| Female | 0.009 (-0.017 to 0.035) | 0.013 | 0.072 (0.049 to 0.095) | 0.012 |
| **Age** |  |  |  |  |
| 80-89 | Reference |  |  |  |
| <60 | -0.018 (-0.073 to 0.037) | 0.028 | -0.031 (-0.108 to 0.045) | 0.039 |
| 60-69 | -0.005 (-0.058 to 0.048) | 0.027 | -0.065 (-0.127 to -0.003) | 0.031 |
| 70-79 | -0.027 (-0.064 to 0.010) | 0.019 | -0.038 (-0.078 to 0.002) | 0.020 |
| 90-99 | -0.018 (-0.063 to 0.028) | 0.023 | -0.017 (-0.061 to 0.028) | 0.023 |
| >100 | -0.237 (-0.423 to -0.050) | 0.095 | -0.014 (-0.198 to 0.170) | 0.094 |
| **Main client group** |  |  |  |  |
| Older adult | Reference |  |  |  |
| Learning disabilities | -0.030 (-0.104 to 0.044) | 0.038 | 0.480 (0.373 to 0.586) | 0.054 |
| Other adult | -0.055 (-0.108 to -0.002) | 0.027 | 0.142 (0.070 to 0.213) | 0.037 |
| **Frailty risk score** |  |  |  |  |
| Low risk (<5) | Reference |  |  |  |
| Intermediate risk (5-15) | -0.020 (-0.059 to 0.020) | 0.020 | 0.015 (-0.021 to 0.051) | 0.019 |
| High risk (>15) | -0.054 (-0.099 to -0.009) | 0.023 | -0.006 (-0.046 to 0.034) | 0.021 |
| **Died during follow up** |  |  |  |  |
| Alive | Reference |  |  |  |
| Dead | 1.038 (0.986 to 1.089) | 0.026 | -0.585 (-0.617 to -0.552) | 0.017 |
| **Interaction: age (year) – Mortality** | | | | |
| 80-89 | Reference |  |  |  |
| <60 | 0.011 (-0.319 to 0.342) | 0.168 | -0.066 (-0.269 to 0.137) | 0.104 |
| 60-69 | 0.317 (0.059 to 0.576) | 0.132 | 0.118 (-0.014 to 0.251) | 0.068 |
| 70-79 | -0.028 (-0.130 to 0.073) | 0.052 | -0.031 (-0.096 to -0.034) | 0.033 |
| 90-99 | 0.021 (-0.061 to 0.103) | 0.042 | -0.023 (-0.075 to 0.030) | 0.027 |
| >100 | 0.253 (-0.102 to 0.607) | 0.181 | -0.040 (-0.255 to 0.176) | 0.110 |
| **Interaction: age (year) - Frailty score, intermediate risk (5-15)** | |  |  |  |
| 80-89 | Reference |  |  |  |
| <60 | 0.027 (-0.052 to 0.106) | 0.040 | 0.011 (-0.110 to 0.132) | 0.062 |
| 60-69 | -0.043 (-0.135 to 0.049) | 0.047 | 0.019 (-0.074 to 0.112) | 0.047 |
| 70-79 | 0.019 (-0.046 to 0.083) | 0.033 | 0.025 (-0.036 to 0.085) | 0.031 |
| 90-99 | 0.045 (-0.027 to 0.117) | 0.037 | 0.013 (-0.048 to 0.074) | 0.031 |
| >100 | 0.369 (0.061 to 0.678) | 0.158 | 0.124 (-0.148 to 0.396) | 0.139 |
| **Interaction: age (year) - Frailty score, high risk (>15)** | | | | |
| 80-89 | Reference |  |  |  |
| <60 | 0.082 (-0.069 to 0.234) | 0.077 | -0.021 (-0.188 to 0.146) | 0.085 |
| 60-69 | 0.082 (-0.051 to 0.214) | 0.068 | -0.076 (-0.196 to 0.043) | 0.061 |
| 70-79 | 0.097 (0.017 to 0.177) | 0.041 | 0.039 (-0.032 to 0.111) | 0.036 |
| 90-99 | 0.037 (-0.044 to 0.118) | 0.041 | 0.033 (-0.035 to 0.102) | 0.035 |
| >100 | 0.140 (-0.284 to 0.564) | 0.216 | 0.088 (-0.234 to 0.409) | 0.164 |

**
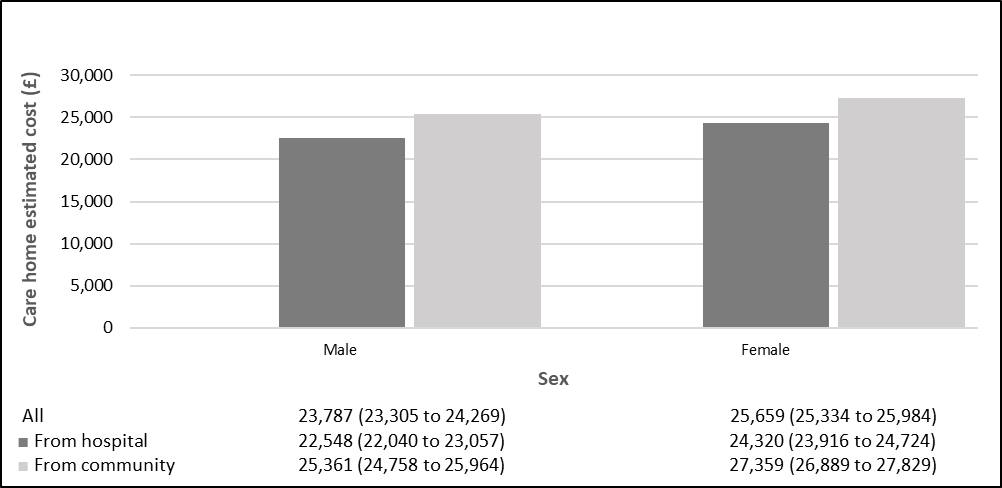
**

**Figure S2. Average annual care-home cost per patient by sex**

**
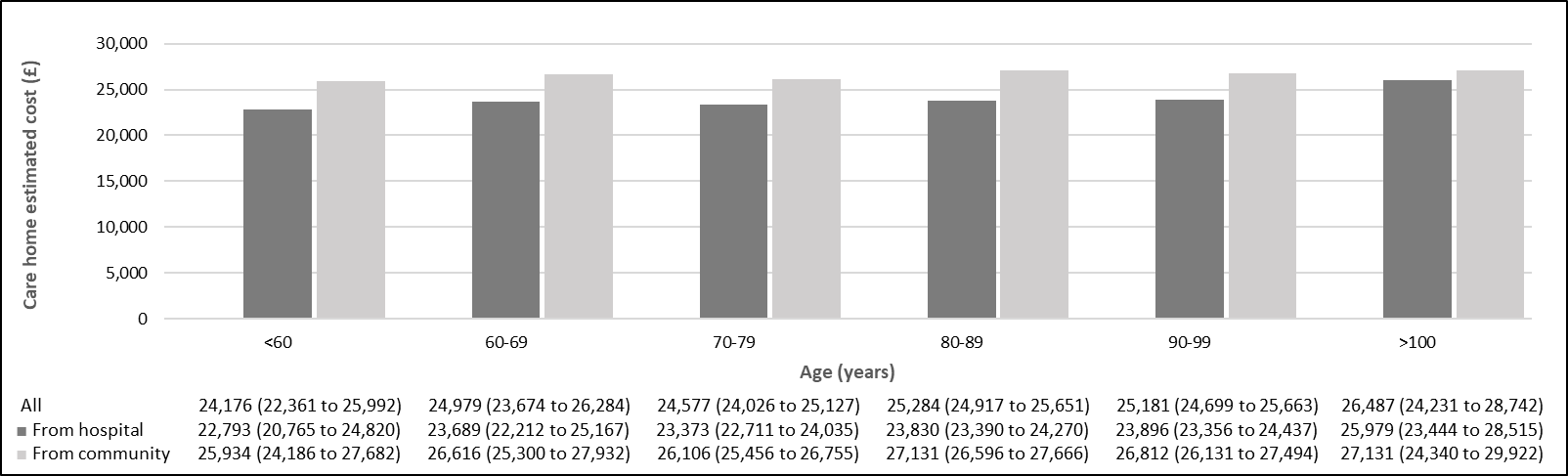
**

**Figure S3. Average annual care-home cost per patient by age**

**
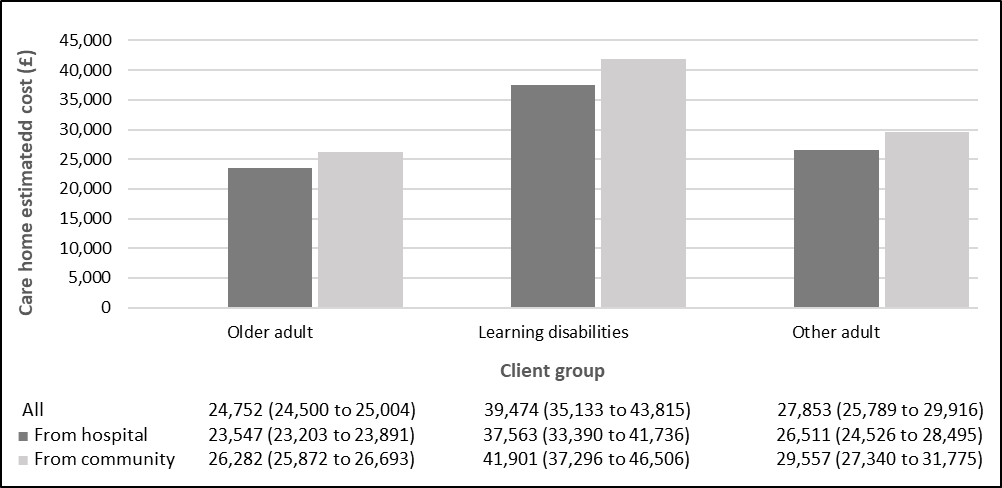
**

**Figure S4. Average annual care-home cost per patient by client group**

**
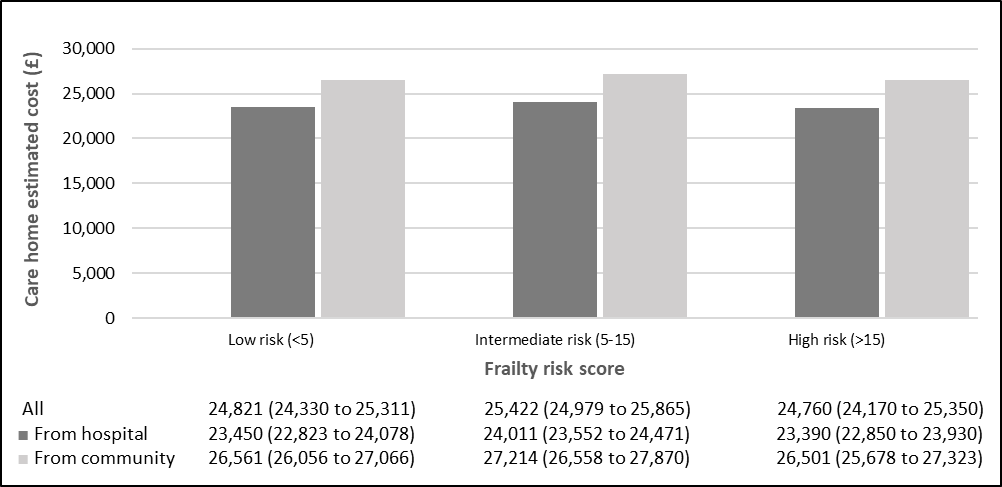
**

**Figure S5. Average annual care-home cost per patient by frailty risk score**

**HOSPITAL COST**

**Table S3. Regression result and interactions for hospital cost**

|  | Probability |  | Cost ratios |  |
| --- | --- | --- | --- | --- |
|  | (First modelling part) |  | (Second modelling part) |  |
| Covariates | Coefficient (95% CI) | SE | Coefficient (95% CI) | SE |
| **Cohort** |  |  |  |  |
| From hospital | Reference |  |  |  |
| From community | -0.059 (-0.146 to 0.028) | 0.044 | 0.034 (-0.047 to 0.114) | 0.041 |
| **Year (cost incurred)** |  |  |  |  |
| 2013/2014 | Reference |  |  |  |
| 2015/2016 | 1.965 (1.864 to 2.066) | 0.051 | 0.145 (0.069 to 0.221) | 0.039 |
| **Sex** |  |  |  |  |
| Male | Reference |  |  |  |
| Female | -0.096 (-0.180 to -0.012) | 0.043 | 0.008 (-0.072 to 0.088) | 0.041 |
| **Age** |  |  |  |  |
| 80-89 | Reference |  |  |  |
| <60 | -0.066 (-0.423 to 0.292) | 0.182 | 0.222 (-0.233 to 0.677) | 0.232 |
| 60-69 | -0.098 (-0.443 to 0.247) | 0.176 | 0.405 (0.022 to 0.787) | 0.195 |
| 70-79 | -0.040 (-0.232 to 0.151) | 0.098 | 0.219 (0.001 to 0.437) | 0.111 |
| 90-99 | -0.057 (-0.254 to 0.139) | 0.100 | -0.097 (-0.292 to 0.098) | 0.100 |
| >100 | -1.616 (-2.362 to -0.870) | 0.381 | -0.485 (-1.456 to 0.486) | 0.495 |
| **Main client group** |  |  |  |  |
| Older adult | Reference |  |  |  |
| Learning disabilities | -0.540 (-0.994 to -0.085) | 0.232 | 0.271 (-0.600 to 1.142) | 0.444 |
| Other adult | 0.273 (-0.054 to 0.599) | 0.167 | 0.260 (-0.104 to 0.624) | 0.186 |
| **Frailty risk score** |  |  |  |  |
| Low risk (<5) | Reference |  |  |  |
| Intermediate risk (5-15) | 0.074 (-0.064 to 0.212) | 0.071 | 0.017 (-0.122 to 0.155) | 0.071 |
| High risk (>15) | 0.197 (0.044 to 0.351) | 0.078 | -0.056 (-0.193 to 0.081) | 0.070 |
| **Died during follow up** |  |  |  |  |
| Alive | Reference |  |  |  |
| Dead | 3.068 (2.949 to 3.187) | 0.061 | -0.057 (-0.170 to 0.056) | 0.058 |
| **Interaction: age (year) – Mortality** | | | | |
| 80-89 | Reference |  |  |  |
| <60 | -0.128 (-0.740 to 0.483) | 0.312 | 0.199 (-0.297 to 0.695) | 0.253 |
| 60-69 | 0.150 (-0.243 to 0.544) | 0.201 | 0.305 (-0.050 to 0.660) | 0.181 |
| 70-79 | -0.099 (-0.297 to 0.100) | 0.101 | 0.032 (-0.183 to 0.247) | 0.110 |
| 90-99 | 0.121 (-0.062 to 0.304) | 0.093 | 0.026 (-0.149 to 0.201) | 0.089 |
| >100 | 0.623 (-0.368 to 1.614) | 0.506 | 0.481 (-0.354 to 0.316) | 0.426 |
| **Interaction: age (year) - Frailty score, intermediate risk (5-15)** | |  |  |  |
| 80-89 | Reference |  |  |  |
| <60 | 0.749 (0.324 to 1.174) | 0.217 | 0.069 (-0.429 to 0.566) | 0.254 |
| 60-69 | 0.352 (-0.083 to 0.786) | 0.222 | -0.432 (-0.870 to 0.005) | 0.223 |
| 70-79 | 0.208 (-0.033 to 0.448) | 0.123 | -0.114 (-0.372 to 0.143) | 0.131 |
| 90-99 | -0.036 (-0.268 to 0.196) | 0.118 | 0.015 (-0.195 to 0.225) | 0.107 |
| >100 | 1.364 (0.442 to 2.285) | 0.470 | 0.182 (-0.623 to 0.988) | 0.411 |
| **Interaction: age (year) - Frailty score, high risk (>15)** | | | | |
| 80-89 | Reference |  |  |  |
| <60 | 1.400 (0.707 to 2.092) | 0.353 | 0.211 (-0.461 to 0.882) | 0.343 |
| 60-69 | 0.221 (-0.291 to 0.732) | 0.261 | -0.102 (-0.588 to 0.384) | 0.248 |
| 70-79 | 0.184 (-0.091 to 0.459) | 0.140 | -0.049 (-0.314 to 0.216) | 0.135 |
| 90-99 | -0.140 (-0.392 to 0.112) | 0.128 | 0.063 (-0.152 to 0.278) | 0.110 |
| >100 | 1.602 (0.439 to 2.766) | 0.593 | -0.037 (-0.773 to 0.699) | 0.376 |

**
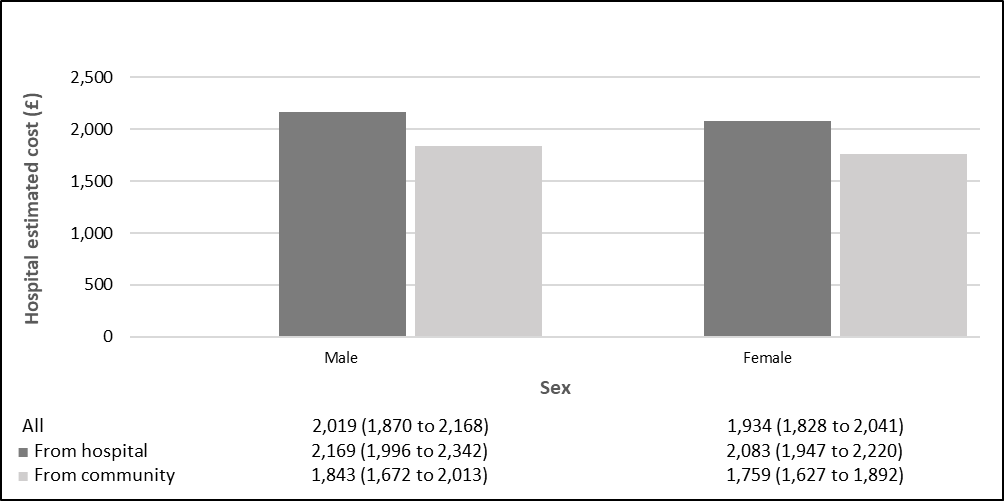
**

**Figure S6. Average annual hospital cost per patient by sex**

**
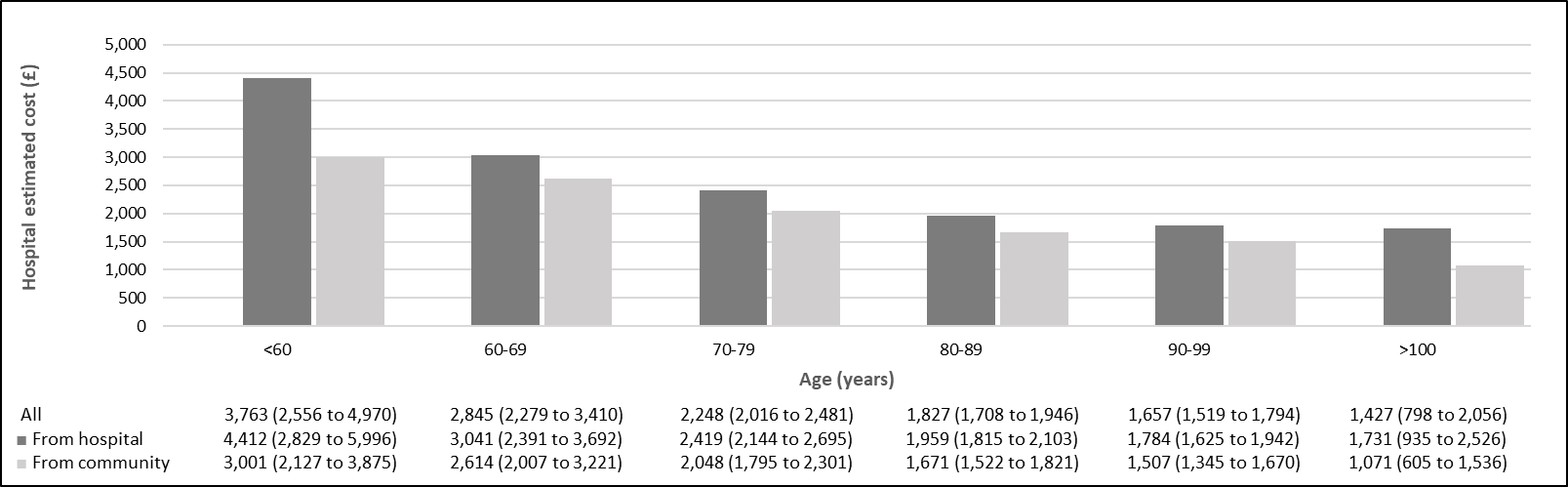
**

**Figure S7. Average annual hospital cost per patient by age**

**
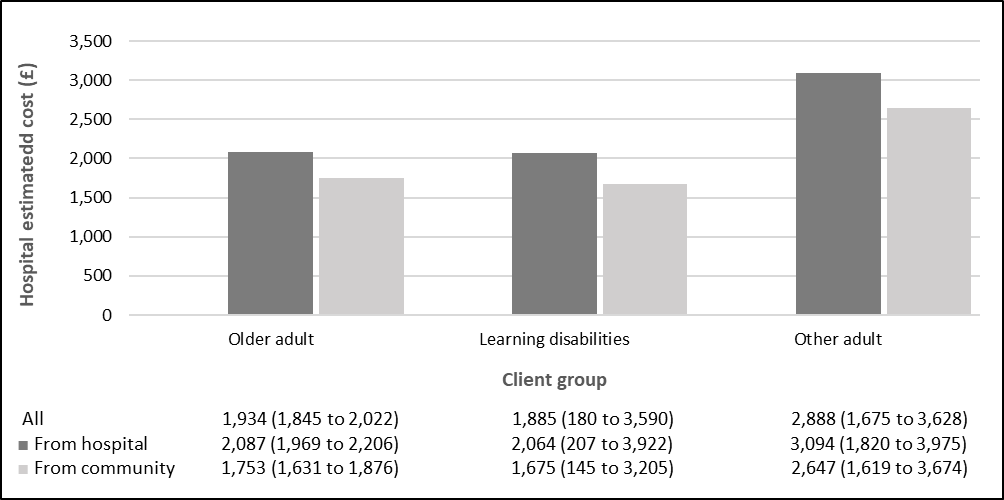
**

**Figure S8. Average annual hospital cost per patient by client group**

**
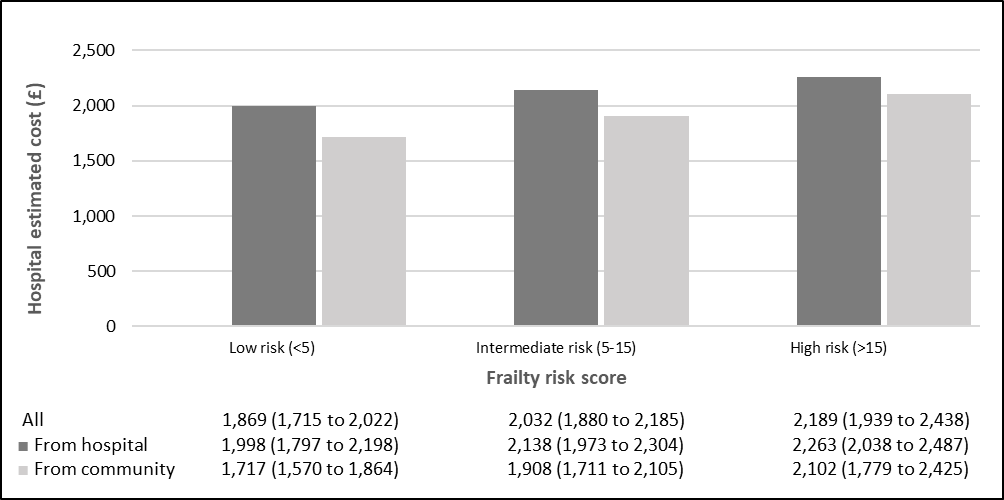
**

**Figure S9. Average annual hospital cost per patient by frailty risk score**

**HOSPITAL COST (year preceding and year after care-home admission)**

**Table S4. Regression result and interactions for hospital cost in the year preceding care-home admission**

|  | Probability |  | Cost ratios |  |
| --- | --- | --- | --- | --- |
|  | (First modelling part) |  | (Second modelling part) |  |
| Covariates | Coefficient (95% CI) | SE | Coefficient (95% CI) | SE |
| **Cohort** |  |  |  |  |
| From hospital | Reference |  |  |  |
| From community | -0.641 (-0.681 to -0.600) | 0.021 | -0.598 (-0.638 to -0.557) | 0.021 |
| **Year (cost incurred)** |  |  |  |  |
| 2012/2013 | Reference |  |  |  |
| 2013/2014 | 1.758 (1.694 to 1.823) | 0.033 | 0.439 (0.388 to 0.489) | 0.026 |
| 2015/2016 | 1.849 (1.772 to 1.927) | 0.039 | 0.689 (0.636 to 0.741) | 0.027 |
| **Sex** |  |  |  |  |
| Male | Reference |  |  |  |
| Female | -0.008 (-0.047 to 0.032) | 0.020 | -0.034 (-0.068 to 0.000) | 0.017 |
| **Age** |  |  |  |  |
| 80-89 | Reference |  |  |  |
| <60 | -0.183 (-0.397 to 0.031) | 0.109 | 0.573 (0.321 to 0.825) | 0.129 |
| 60-69 | 0.088 (-0.097 to 0.272) | 0.094 | 0.483 (0.161 to 0.804) | 0.164 |
| 70-79 | -0.119 (-0.231 to -0.007) | 0.057 | 0.098 (-0.022 to 0.218) | 0.061 |
| 90-99 | 0.036 (-0.072 to 0.144) | 0.055 | -0.221 (-0.343 to -0.099) | 0.062 |
| >100 | -0.283 (-0.888 to 0.322) | 0.309 | -0.766 (-1.199 to -0.333) | 0.221 |
| **Main client group** |  |  |  |  |
| Older adult | Reference |  |  |  |
| Learning disabilities | -0.550 (-0.808 to -0.292) | 0.132 | 0.494 (0.007 to 0.980) | 0.248 |
| Other adult | 0.210 (0.046 to 0.374) | 0.084 | -0.207 (-0.354 to -0.060) | 0.075 |
| **Frailty risk score** |  |  |  |  |
| Low risk (<5) | Reference |  |  |  |
| Intermediate risk (5-15) | 0.863 (0.791 to 0.935) | 0.037 | 0.026 (-0.051 to 0.102) | 0.039 |
| High risk (>15) | 1.123 (1.045 to 1.202) | 0.040 | 0.135 (0.059 to 0.211) | 0.039 |
| **Died during follow up** |  |  |  |  |
| Alive | Reference |  |  |  |
| Dead | 1.086 (1.027 to 1.146) | 0.030 | 0.028 (-0.017 to 0.072) | 0.023 |
| **Interaction: age (year) – Mortality** | | | | |
| 80-89 | Reference |  |  |  |
| <60 | 0.550 (0.197 to 0.903) | 0.180 | -0.158 (-0.441 to 0.125) | 0.144 |
| 60-69 | 0.394 (0.141 to 0.647) | 0.129 | -0.106 (-0.285 to 0.073) | 0.091 |
| 70-79 | 0.133 (0.024 to 0.241) | 0.056 | 0.000 (-0.087 to 0.88) | 0.045 |
| 90-99 | 0.003 (-0.088 to 0.094) | 0.046 | -0.002 (-0.075 to 0.171) | 0.037 |
| >100 | 0.535 (0.163 to 0.908) | 0.190 | -0.001 (-0.318 to 0.115) | 0.162 |
| **Interaction: age (year) - Frailty score, intermediate risk (5-15)** | |  |  |  |
| 80-89 | Reference |  |  |  |
| <60 | 0.246 (0.013 to 0.479) | 0.119 | -0.022 (-0.318 to 0.173) | 0.151 |
| 60-69 | -0.048 (-0.268 to 0.171) | 0.112 | -0.132 (-0.454 to 0.190) | 0.164 |
| 70-79 | 0.082 (-0.046 to 0.210) | 0.065 | 0.046 (-0.086 to 0.78) | 0.067 |
| 90-99 | -0.031 (-0.149 to 0.088) | 0.061 | 0.140 (0.011 to 0.169) | 0.066 |
| >100 | -0.101 (-0.733 to 0.531) | 0.322 | 0.670 (0.231 to 0.109) | 0.224 |
| **Interaction: age (year) - Frailty score, high risk (>15)** | | | | |
| 80-89 | Reference |  |  |  |
| <60 | 0.557 (0.218 to 0.896) | 0.173 | -0.065 (-0.366 to 0.237) | 0.154 |
| 60-69 | -0.017 (-0.273 to 0.239) | 0.130 | -0.027 (-0.360 to 0.105) | 0.170 |
| 70-79 | 0.184 (0.042 to 0.326) | 0.072 | -0.050 (-0.182 to 0.082) | 0.067 |
| 90-99 | -0.036 (-0.166 to 0.094) | 0.066 | 0.140 (0.011 to 0.269) | 0.066 |
| >100 | -0.271 (-0.919 to 0.377) | 0.331 | 0.668 (0.247 to 0.190) | 0.215 |

**Table S5. Regression result and interactions for hospital cost in the year after care-home admission**

|  | Probability |  | Cost ratios |  |
| --- | --- | --- | --- | --- |
|  | (First modelling part) |  | (Second modelling part) |  |
| Covariates | Coefficient (95% CI) | SE | Coefficient (95% CI) | SE |
| **Cohort** |  |  |  |  |
| From hospital | Reference |  |  |  |
| From community | -0.039 (-0.129 to 0.050) | 0.046 | 0.073 (-0.012 to 0.158) | 0.043 |
| **Year (cost incurred)** |  |  |  |  |
| 2013/2014 | Reference |  |  |  |
| 2015/2016 | 1.623 (1.518 to 1.727) | 0.053 | 0.103 (0.024 to 0.183) | 0.041 |
| **Sex** |  |  |  |  |
| Male | Reference |  |  |  |
| Female | -0.077 (-0.163 to 0.009) | 0.044 | -0.013 (-0.095 to 0.068) | 0.041 |
| **Age** |  |  |  |  |
| 80-89 | Reference |  |  |  |
| <60 | -0.081 (-0.444 to 0.282) | 0.185 | 0.303 (-0.187 to 0.793) | 0.250 |
| 60-69 | -0.135 (-0.494 to 0.225) | 0.183 | 0.372 (-0.069 to 0.813) | 0.225 |
| 70-79 | -0.105 (-0.304 to 0.093) | 0.101 | 0.169 (-0.065 to 0.403) | 0.119 |
| 90-99 | -0.085 (-0.286 to 0.116) | 0.103 | -0.127 (-0.340 to 0.086) | 0.109 |
| >100 | -1.446 (-2.190 to -0.701) | 0.380 | -0.419 (-1.440 to 0.602) | 0.521 |
| **Main client group** |  |  |  |  |
| Older adult | Reference |  |  |  |
| Learning disabilities | -0.791 (-1.286 to -0.296) | 0.252 | 0.411 (-0.669 to 1.491) | 0.551 |
| Other adult | 0.090 (-0.239 to 0.419) | 0.168 | 0.260 (-0.154 to 0.674) | 0.211 |
| **Frailty risk score** |  |  |  |  |
| Low risk (<5) | Reference |  |  |  |
| Intermediate risk (5-15) | 0.013 (-0.129 to 0.156) | 0.073 | -0.022 (-0.170 to 0.126) | 0.075 |
| High risk (>15) | 0.161 (0.002 to 0.320) | 0.081 | -0.116 (-0.265 to 0.033) | 0.076 |
| **Died during follow up** |  |  |  |  |
| Alive | Reference |  |  |  |
| Dead | 3.172 (3.052 to 3.292) | 0.061 | -0.028 (-0.142 to 0.086) | 0.058 |
| **Interaction: age (year) – Mortality** | | | | |
| 80-89 | Reference |  |  |  |
| <60 | -0.230 (-0.809 to 0.350) | 0.296 | 0.223 (-0.302 to 0.748) | 0.268 |
| 60-69 | 0.258 (-0.153 to 0.669) | 0.210 | 0.225 (-0.167 to 0.616) | 0.200 |
| 70-79 | -0.042 (-0.247 to 0.164) | 0.105 | 0.077 (-0.144 to 0.299) | 0.113 |
| 90-99 | 0.122 (-0.066 to 0.311) | 0.096 | -0.023 (-0.207 to 0.162) | 0.094 |
| >100 | 0.289 (-0.694 to 1.272) | 0.502 | 0.444 (-0.398 to 1.286) | 0.430 |
| **Interaction: age (year) - Frailty score, intermediate risk (5-15)** | |  |  |  |
| 80-89 | Reference |  |  |  |
| <60 | 0.855 (0.421 to 1.289) | 0.221 | -0.009 (-0.572 to 0.553) | 0.287 |
| 60-69 | 0.296 (-0.158 to 0.749) | 0.231 | -0.358 (-0.861 to 0.144) | 0.256 |
| 70-79 | 0.213 (-0.035 to 0.461) | 0.127 | -0.110 (-0.379 to 0.158) | 0.137 |
| 90-99 | 0.005 (-0.233 to 0.242) | 0.121 | 0.104 (-0.121 to 0.329) | 0.115 |
| >100 | 1.413 (0.484 to 2.342) | 0.474 | 0.162 (-0.694 to 1.019) | 0.437 |
| **Interaction: age (year) - Frailty score, high risk (>15)** | | | | |
| 80-89 | Reference |  |  |  |
| <60 | 1.359 (0.725 to 1.993) | 0.323 | -0.143 (-0.683 to 0.397) | 0.276 |
| 60-69 | 0.243 (-0.260 to 0.746) | 0.257 | 0.102 (-0.437 to 0.641) | 0.275 |
| 70-79 | 0.208 (-0.076 to 0.491) | 0.145 | -0.046 (-0.332 to 0.241) | 0.146 |
| 90-99 | -0.147 (-0.404 to 0.110) | 0.131 | 0.138 (-0.095 to 0.372) | 0.119 |
| >100 | 1.623 (0.447 to 2.800) | 0.600 | -0.006 (-0.826 to 0.813) | 0.418 |

**
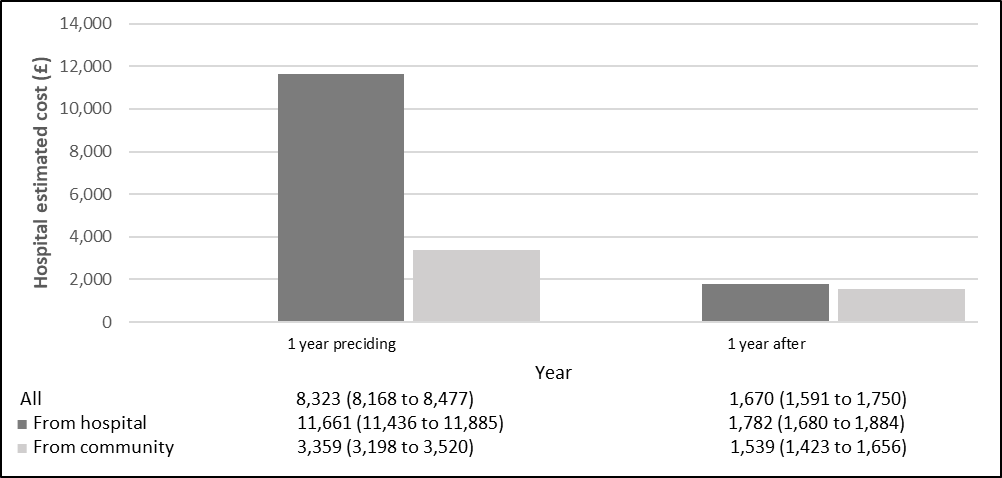
**

**Figure S10. Hospital cost incurred in the year preceding and the year after care-home admission**
